# Transcriptome-Wide Identification and Integrated Analysis of a *UGT* Gene Involved in Ginsenoside Ro Biosynthesis in *Panax ginseng*

**DOI:** 10.3390/plants13050604

**Published:** 2024-02-23

**Authors:** Xiaochen Yu, Jinghui Yu, Sizhang Liu, Mingming Liu, Kangyu Wang, Mingzhu Zhao, Yanfang Wang, Ping Chen, Jun Lei, Yi Wang, Meiping Zhang

**Affiliations:** 1 College of Life Science, Jilin Agricultural University, Changchun 130118, China; yu-xiaochen@foxmail.com (X.Y.); jinghui-yu@foxmail.com (J.Y.); lsz512411412@163.com (S.L.); lmm19971107@163.com (M.L.); kangyu.wang@jlau.edu.cn (K.W.); mingzhuzhao@jlau.edu.cn (M.Z.); chenping201407@126.com (P.C.); leijun3000@jlau.edu.cn (J.L.); 2 Jilin Engineering Research Center Ginseng Genetic Resources Development and Utilization, Changchun 130118, China; 3 College of Chinese Medicinal Materials, Jilin Agricultural University, Changchun 130118, China; yfwang2014@163.com

**Keywords:** *Panax ginseng*, ginsenoside Ro, transcriptome, glycosyltransferase, biosynthesis

## Abstract

*Panax ginseng* as a traditional medicinal plant with a long history of medicinal use. Ginsenoside Ro is the only oleanane-type ginsenoside in ginseng, and has various pharmacological activities, including anti-inflammatory, detoxification, and antithrombotic activities. UDP-dependent glycosyltransferase (UGT) plays a key role in the synthesis of ginsenoside, and the excavation of UGT genes involved in the biosynthesis of ginsenoside Ro has great significance in enriching ginsenoside genetic resources and further revealing the synthesis mechanism of ginsenoside. In this work, ginsenoside-Ro-synthesis-related genes were mined using the *P. ginseng* reference-free transcriptome database. Fourteen hub transcripts were identified by differential expression analysis and weighted gene co-expression network analysis. Phylogenetic and synteny block analyses of *PgUGAT252645*, a *UGT* transcript among the hub transcripts, showed that *PgUGAT252645* belonged to the *UGT73* subfamily and was relatively conserved in ginseng plants. Functional analysis showed that *PgUGAT252645* encodes a glucuronosyltransferase that catalyzes the glucuronide modification of the C3 position of oleanolic acid using uridine diphosphate glucuronide as the substrate. Furthermore, the mutation at 622 bp of its open reading frame resulted in amino acid substitutions that may significantly affect the catalytic activity of the enzyme, and, as a consequence, affect the biosynthesis of ginsenoside Ro. Results of the in vitro enzyme activity assay of the heterologous expression product in *E. coli* of *PgUGAT252645* verified the above analyses. The function of *PgUGAT252645* was further verified by the result that its overexpression in ginseng adventitious roots significantly increased the content of ginsenoside Ro. The present work identified a new *UGT* gene involved in the biosynthesis of ginsenoside Ro, which not only enriches the functional genes in the ginsenoside synthesis pathway, but also provides the technical basis and theoretical basis for the in-depth excavation of ginsenoside-synthesis-related genes.

## 1. Introduction

*Panax ginseng* C. A. Meyer (*P. ginseng*), a perennial herb of the Ginseng genus (*Panax* L.) in the *Araliaceae* family, is a valuable traditional Chinese herb that is widely used worldwide for the clinical treatment of many diseases and for the production of health food products due to its high medicinal value [1]. Ginsenosides are a group of secondary metabolites of plants of the Ginseng genus and are the main medicinal active ingredients of *P. ginseng* [2]. The different types of triterpene molecules, glycosylation sites, and the number of glycosyl groups in the molecular structure of various ginsenosides determine their diverse pharmacological activities [3,4,5].

**Figure 1:**
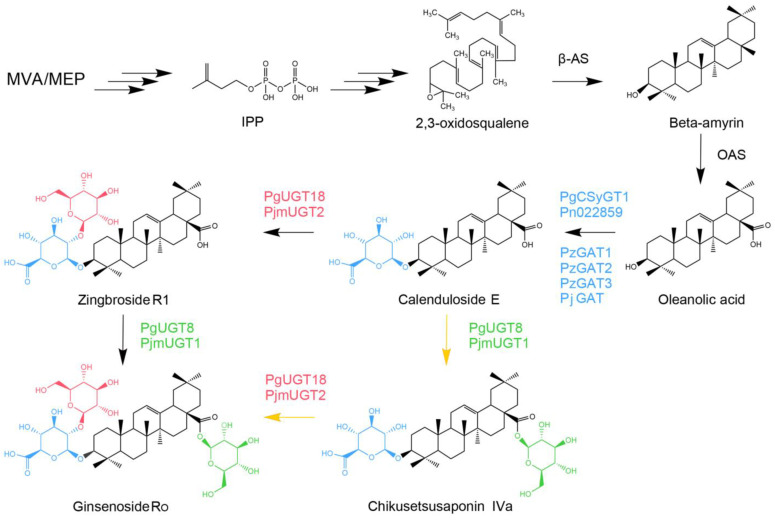
Biosynthetic pathway of ginsenoside Ro. Up to the oleanolic acid synthase, key enzymes on this synthetic pathway have been cloned and characterized in several species. The reported glycosyltransferases from different plants of the Ginseng genus directly involved in the synthesis of ginsenoside Ro are marked with different colors, and the relevant glycosyl groups in the glycosylation modification steps they catalyze are marked with the corresponding colors. Yellow arrows mark the main glycosylation modification pathways.

Ginsenoside Ro is the only reported oleanane-type pentacyclic triterpenoid ginsenoside of *P. ginseng* origin [6] with known pharmacological activities including the inhibition of tumor growth [7], antithrombotic activity [8], anti-inflammatory activity [9], and the improvement of obesity [10]. It has been reported that, similar to the ginsenosides Rb1 and Re that are involved in regulating the growth of *P. ginseng* adventitious root [11], ginsenoside Ro has a low concentration-promoting and high concentration-inhibiting effect on the growth of *P. ginseng* adventitious roots [12]. The synthetic pathway of Ginsenoside Ro is identical to that of tetracyclic triterpenoid ginsenosides before the synthesis of its precursor beta-amyrin, both of which use the 2,3-oxysqualene catalytically generated from the end product of the mevalonate (MVA) and 2-c-methyl-d-erythritol 4-phosphate (MEP) pathways as the substrate for the synthesis of the triterpene molecular skeleton [13,14]. Ginsenoside Ro is a glycosylation modification product of oleanolic acid, which is catalyzed from beta-amyrin by oleanolic acid synthase. This includes glucuronidation at the C3 position of oleanolic acid, glycosylation at the C2 position of this glucuronide group, and glycosylation at the C28 position of Zingbroside R1 or Chikusetsusaponin IVa (Figure 1).

Studies on genes related to ginsenoside Ro synthesis are still scarce, and currently only include the cloning and characterization of synthase genes for precursors such as β-amyrin [15] and oleanolic acid [16], as well as a group of glycosyltransferases belonging to different gene families derived from *Panax ginseng* [17], *Panax notoginseng* [18], *Panax zingiberensis* [19] and *Panax japonicus* [20], which have been shown to be involved in the glycosylation modification step of ginsenoside Ro synthesis. However, due to the complexity of the metabolic network, many key genes and regulatory factors associated with this synthetic pathway remain unexplored. Therefore, it is of great importance to continue to explore the genes related to ginsenoside Ro synthesis in order to enrich *P. ginseng* genetic resources and further reveal the mechanism of ginsenoside synthesis.

In this work, we assembled a reference-free transcriptome of four-year old *P. ginseng* main roots and performed a series of systematic analyses with reference to the ginsenoside Ro content of each sample. These analyses included correlation analysis between the expression of transcripts within the transcriptome and the ginsenoside Ro content of the samples, differential expression analysis of transcripts between samples with higher and lower ginsenoside Ro content and weighted gene co-expression network analysis of candidate transcripts. From these analyses, we identified 14 hub transcripts, including three reported glycosyltransferase transcripts directly involved in ginsenoside Ro synthesis. In addition, results of SNPs/InDels mining and molecular docking analyses indicated that *PgUGAT252645* might be a transcript of the *P. ginseng* UDP-glucuronosyltransferase directly involved in ginsenoside Ro synthesis. Thus, the functional validation of *PgUGAT252645* was performed by in vitro activity assay and the overexpression achieved by Agrobacterium-mediated genetic transformation of *P. ginseng* adventitious roots.

## 2. Materials and Methods

### 2.1. Database and Plant Materials

RNA sequencing data from 344 4-year-old *P. ginseng* main root samples were used to assemble the transcriptome. The datasets can be found in online repositories (https://www.ncbi.nlm.nih.gov/bioproject/PRJNA302556 (accessed on 8 August 2023)) [21]. Data of the ginsenosides’ content of these samples were used for subsequent studies. The ginseng adventitious roots used in this work were derived from callus induction in a previous laboratory [22] and cultured in a B5 medium for expansion.

### 2.2. Strains and Vectors

*E. coli* DH5α containing plasmid pET-28a and the chemically competent cells of *E. coli* BL21 (DE3) were purchased from Sangon Biotech Co., Ltd. (Shanghai, China). The chemically competent cells of *Ag. rhizogenes* C58C1 were purchased from Huayueyang Biotech Co., Ltd., Beijing, China. Plasmid pCAMBIA1300-35S was purchased from MiaoLing Plasmid Platform, Wuhan, China. The DNA fragment of the open reading frame of the target transcript was synthesized by Comate Bioscience Co., Ltd., Jilin, China.

### 2.3. Assemble of the Reference-Free Transcriptome

The reference-free transcriptome was assembled using RNA sequencing data from 344 four-year-old *P. ginseng* main root samples by Trinity version 2.14.0 [23] using the reference-free transcriptome as the reference; expressions of transcripts in count and transcripts per million (TPM) were calculated by Hisat2 version 2.0.5 [24].

### 2.4. Correlation-Based Mining of Transcripts

It has been shown that the expression of a transcript controlling a quantitative trait is always highly correlated with the phenotype of the trait it controls [25]. Therefore, in order to initially explore the genes associated with ginsenoside Ro synthesis, 30 samples with the highest ginsenoside Ro content and 30 samples with the lowest ginsenoside Ro content were selected from the 344 samples and divided into high and low ginsenoside Ro content groups. The expression (TPM) of each transcript within the transcriptome in these 60 samples was quantified. Spearman correlation analysis of expression with ginsenoside Ro content was then performed. If the expression of a transcript correlated with the ginsenoside Ro content at a significance level of *p* ≤ 0.01, it was tentatively concluded that the transcript was involved in the biosynthesis of Ro.

### 2.5. Analysis of Differentially Expressed Transcripts

Differentially expressed transcripts between the high- and low-Ro-content groups were analyzed using the DEseq2 program package [26] in R version 4.2.0 with the ginsenoside Ro-content data of the above grouped samples and the expression (count) of each transcript in the samples in the groups; the transcript with differential expression (FDR ≤ 0.1) between samples in the high- and low-Ro-content groups were defined as differentially expressed transcripts.

### 2.6. Functional Annotation of Candidate Transcripts

Differentially expressed transcripts that showed a significant positive correlation between their expression (TPM) and Ro content were defined as candidate transcripts. These candidate transcripts were functionally annotated in gene ontology (GO) using the Blast2go version 6.0.3 [27], and the annotation results were classified and counted by function at level 2 of the GO.

### 2.7. Weighted Gene Co-Expression Network Analysis (WGCNA) of Candidate Transcripts

The relationship between candidate transcripts and the ginsenoside Ro content of samples was further analyzed by constructing a weighted co-expression network. Using the expression (TPM) of each candidate transcript in each sample as input data, the co-expression network was constructed by using the WGCNA program package [28] in R version 4.2.0. Correlation between these candidate transcripts were calculated using the Spearman correlation matrix, and soft threshold values were calculated using network topology analysis. The original matrix was transformed to obtain the adjacency matrix after selecting the appropriate soft threshold. Then, the adjacency matrix was transformed into a topological overlap matrix, and the dynamic cut method was applied to cluster the genes and divide the modules. The minimum number of transcripts within the module was set to 30 and the similar module merging threshold was 0.85. The expression pattern of each transcript within the module in each sample was presented by module eigenvalues, and the relationship between the module and ginsenoside Ro content was determined by module eigenvector analysis.

### 2.8. Visualization of Co-Expression Network and Mining of Hub Transcripts

To further filter the candidate transcripts within the co-expression network, we used the Cytoscape version 3.9.1 [29] to visualize the co-expression relationships among the candidate transcripts within the six modules, with each node in the co-expression network representing one candidate transcript and the connecting lines representing the relationships among the candidate transcripts. Hub transcripts within the high weight interval were selected using the weight values as indicators. Then, for validation of these hub transcripts, the expression (TPM) of the reported key enzyme transcripts directly involved in ginsenoside Ro synthesis and hub transcripts were used to build a Spearman correlation coefficient-based interaction network.

### 2.9. Synteny Block and Phylogenetic Analysis of Uridine Diphosphate Glycosyltransferases (UGT) Transcripts in Hub Transcripts

The open reading frame (ORF) region of UGT transcripts in hub transcripts was identified using the ORF Finder of the NCBI website. They were used as query sequences for BLAST in the genomes of several plants from *Panax* L. [30,31] for synteny block analysis (Table S6). The amino acid sequences they encoded and those reported *UGTs* from different plants capable of glycosylating modified pentacyclic triterpenes (Table S6) were aligned with ClustalW, and the phylogenetic tree was constructed using the neighbor-joining method with default parameters and 1000 bootstrap replications in MEGA 7 software [32].

### 2.10. Analysis on the effect of SNPs/InDels of UGT Gene Family on the Content of Ginsenoside Ro in P. ginseng

Multiple *UGT* mRNA sequences from different plants were downloaded from the NCBI website and used as interrogated sequences or Blastn comparisons in the reference-free transcriptome. In addition, the hidden Markov model of UGT conserved structural domains downloaded from the PFAM website was also used to interrogate UGT amino acid sequences in the amino acid sequence database translated from the reference-free transcriptome. The above query results were screened and retrieved, and the resulting transcripts were combined into a single collection, which was used as a reference genome for mining SNPs/InDels in the *UGT* gene family of *P. ginseng*. The SNPs/InDels with a significant effect (*p* ≤ 0.05) on the content of ginsenoside Ro were screened by the Student’s *t*-test using SPSS package (IBM (Armonk, NY, USA) SPSS Statistics version 23.0).

### 2.11. Molecular Docking Analysis of Target UGT Transcript-Encoded Enzyme Proteins with Sugar Donors

Amino acid sequences encoded by the open reading frame regions of the target UGT transcript were subjected to online protein modelling using the SWISS-MODEL website. Online molecular docking analysis of the protein model as well as the sugar donors was then performed using the SwissDock website, and the docking results were visualized and further analyzed using the UCSF chimera, version 1.13.1.

### 2.12. In Vitro Expression and Activity Assay of Target UGT Transcript

The recombinant plasmid for in vitro expression, pET-28a-UGAT, was constructed by ligating the synthetic fragment of the ORF of the target *UGAT* transcripts to the position between the enzymatic cleavage sites *BamH* I and *Hind* III of plasmid pET-28a by seamless cloning. The recombinant plasmid was then transferred into the chemically competent cell of *E. coli* BL21 (DE3) to construct the strain BL21-UGAT for in vitro expression.

Recombinant strain BL21-UGAT cells were cultured at 37 °C, 180 rpm until the OD600 reached 0.8. Then, IPTG with a final concentration of 0.1 mM was added, and protein expression was continuously induced at 16 °C, 180 rpm, for 12 h. The cells were collected by centrifugation at 4 °C, 12,000 rpm for 15 min and then resuspended with HEPES solution. Then, the cells were ultrasonically broken. The supernatant was collected by centrifugation at 4 °C, 12,000 rpm for 45 min, and it was used for the enzyme activity assay.

A volume of 100 μL of the enzyme activity assay reaction system consisted of 85 μL of the above supernatant, 0.5 mM acceptor substrate, and 5 mM glycosyl donor substrate. After incubation at 30 °C for 12 h, the reaction was terminated by adding an equal volume of water-saturated n-butanol, followed by separation of the organic phase and evaporation to dryness. The residue was dissolved in chromatographic grade methanol and analyzed by LC-MS under the conditions described in the Table S3.

### 2.13. Induction and Culture of Ginseng Hairy Roots Overexpressing Target UGT Transcript

Referring to reports on the validation of the function of key genes for ginsenoside synthesis in *P. ginseng* by means of overexpression [33,34], the recombinant plasmid was constructed by ligating the synthetic fragments of the open reading frame of target *UGAT* transcripts to the position between the enzymatic cleavage sites *Sac* I and *Sal* I of pCAMBIA1300-35S by seamless cloning. The recombinant plasmid was then transformed into *Ag. rhizogenes* C58C1 chemically competent cells, which were then screened on a YEP solid medium containing rifampicin, kanamycin, and streptomycin to obtain positive strains. The positive strains were cultured in a YEP liquid medium containing the above antibiotics to OD_600_ = 0.4. The cells were then collected by centrifugation and resuspended in liquid 1/2 MS medium containing acetosyringone (AS) to OD_600_ = 0.5. Pre-cultured ginseng adventitious roots were added to the bacterial suspension for infestation and the infested adventitious roots were first placed on a solid 1/2 MS medium containing 20 μM AS and then transferred to a 1/2 MS medium containing cephalexin until hairy roots developed. Then, each asexual single root line, arising from the successor culture, was expanded in shake flasks using liquid 1/2 MS medium (at 22 °C, 110 rpm). A portion of the recovered culture was immediately frozen in liquid nitrogen and stored at −80 °C to extract genomic DNA for PCR and total RNA for quantitative real-time PCR (qRT-PCR), and a portion was dried to a constant weight and stored at 4 °C for the extraction of ginsenosides.

### 2.14. Extraction and Subsequent Analysis of Ginseng Hairy Roots Overexpressing Target UGT Transcript

Genomic DNA was extracted from three biological replicates of each frozen asexual single root line using the cetyltrimethylammonium bromide (CTAB) plant DNA extraction method. PCR amplification of the specific fragment was then performed using it as a template to verify the successful transfer of the overexpressing fragment to screen for overexpressing-positive hairy root asexual root lines. Total RNA was isolated from three biological replicates of each overexpressing-positive hairy root asexual root line by using the TRIpure Reagent Total RNA Extraction Reagent from BioTeke, Beijing, China. and reverse-transcribed into cDNA using the HiFiScript gDNA Removal cDNA Synthesis Kit from CWBIO, Taizhou, China. The transcript level of the target transcript in each sample was measured by qPCR in ABI 7500 Fast Real-Time PCR System with *Actin 1* as the internal reference gene [35] using UltraSYBR Mixture (Low ROX) from Jiangsu Cowin Biotech Co., Taizhou, China (Tables S2 and S4). Three technical replicates were performed for each of these samples.

### 2.15. Extraction, Detection and Analysis of Ginsenosides

Ginsenosides were extracted by Soxhlet extraction from dried samples of three biological replicates of each overexpressing-positive hairy root asexual single root line. An amount of 0.1 g of each sample was used for ginsenoside extraction. Three technical replicates were performed in each sample. Extractions were analyzed by LC-MS under the conditions described in the Table S5.

## 3. Results

### 3.1. Assembly of the Reference-Free Transcriptome

The reference-free transcriptome assembled by the above method was 3.2 GB in size and included more than 1.5 million transcripts with an average length of 2100 bp (Figure S1); the ratio of the sum of guanine (G) and cytosine (C) bases in the RNA sequence was 43%.

### 3.2. Correlation-Based Mining of Transcripts

**Figure 2:**
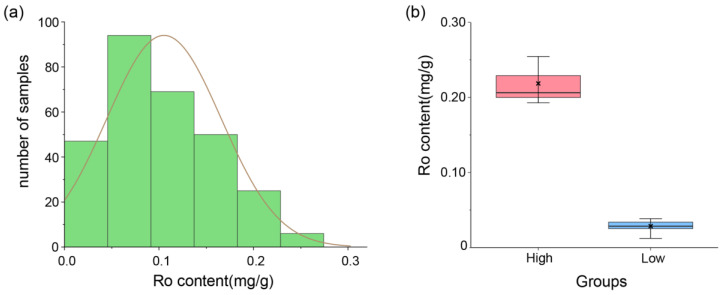
Ginsenoside Ro content in ginseng samples: (**a**) normal distribution plot of ginsenoside Ro content for 344 samples. Ginsenoside Ro content of all samples follows a normal distribution, indicating significant differences among certain samples; and (**b**) grouping of samples with significant differences in ginsenoside Ro content. The pink box represents the high-content group, and the range of ginsenoside Ro content of the samples within the group was 0.187–0.256 mg/g, with a mean value of 0.211 mg/g. The blue box represents the low-content group, and the range of ginsenoside Ro content of the samples within the group was 0.012–0.037 mg/g, with a mean value of 0.027 mg/g. This indicates that there is a significant difference in the content of ginsenoside Ro between the two groups.

Significant differences in ginsenoside Ro content were found between samples of the high and low Ro content groups that were selected from the 344 samples (Figure 2). Spearman correlation analysis of the expression (TPM) of each transcript in the reference-free transcriptome with the ginsenoside Ro content in these samples showed that a total of 22,481 transcripts showed significant positive correlation with the Ro content when the significance level was *p* ≤ 0.01.

### 3.3. Analysis of Differentially Expressed Transcripts

**Figure 3:**
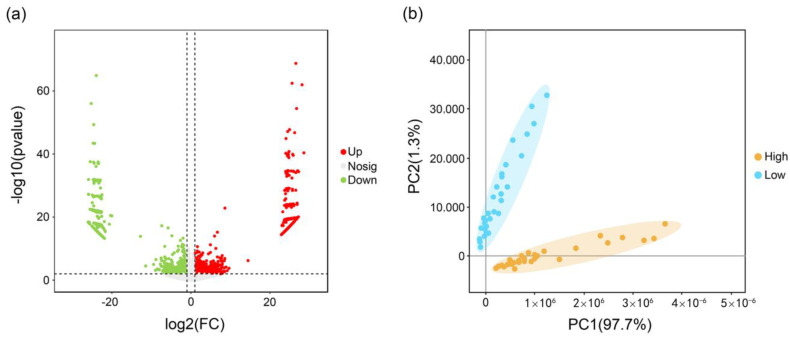
Analysis of differential expression transcripts: (**a**) volcano plot of differentially expressed transcripts. Red dots represent upregulated transcripts, green dots represent downregulated transcripts, and grey dots represent non-differential transcripts; and (**b**) principal component analysis of differential expression transcripts. Blue dots represent low-content group samples, yellow dots represent high-content group samples. Confidence ellipses are marked with corresponding colors.

The results of the differential expression analysis showed that a total of 2014 transcripts were defined as differentially expressed transcripts, with a total of 1236 upregulated transcripts and 778 downregulated transcripts (Figure 3a). The results of the principal component analysis (PCA) showed that there were significant differences in the expression (count) of these differentially expressed transcripts between these two groups of samples (Figure 3b).

### 3.4. Functional Annotation of Candidate Transcripts

**Figure 4:**
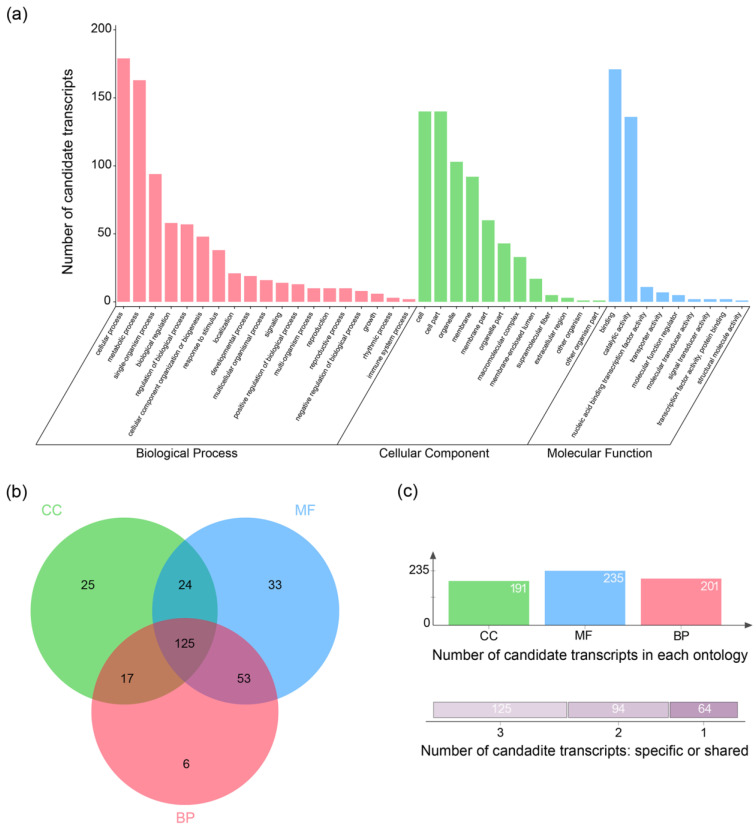
Functional annotation of candidate transcripts: (**a**) histogram of the distribution of candidate transcripts at level 2 terms. The height of the column indicates the number of transcripts annotated to the level 2 term: (**b**) Wayne diagrams for functional annotation of candidate transcripts; and (**c**) histogram of ontology distribution of candidate transcripts. Green columns represent cellular component, blue columns represent molecular function, and pink columns represent biological process.

A total of 846 transcripts were defined as candidate transcripts based on the definition approach described previously. After classifying and counting the function annotation results of these candidate transcripts, we found that the functions of the annotated candidate transcripts were distributed within each of the three main ontological entries in GO, and were more numerous in some level 2 GO terms, such as the cellular process (179), metabolic process (163), single-organism process (94), binding (171), catalytic activity (136), cell (140), cell part (140), organelle (103) and membrane (92) (Figure 4a), with a total of 125 candidate transcripts having functional annotation results for all three ontologies at the same time (Figure 4c).

### 3.5. Weighted Gene Co-Expression Network Analysis (WGCNA) of Candidate Transcripts

**Figure 5:**
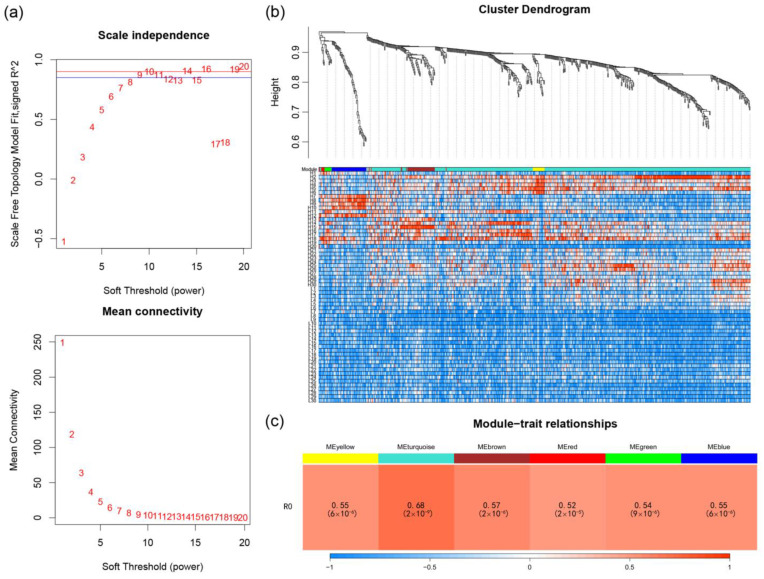
Construction of weighted gene co-expression network: (**a**) determination of soft thresholds; (**b**) shearing and merging of gene topology overlapping clustering trees and delineation of gene modules; and (**c**) heatmap of association between gene modules and ginsenoside Ro content.

In order to make the weight co-expression network conform to the scale-free network distribution, we used the Pick Soft Threshold function to calculate and determine the optimal soft threshold value. The calculation results show that when the soft threshold value β = 10, the scale-free network fit index R^2^ > 0.90 and the average connectivity tended to 0. Therefore, a soft threshold value β = 10 was determined to construct the weight co-expression network (Figure 5a).

After determining the soft threshold, we sheared and merged the gene topology overlap clustering trees that we constructed (Figure 5b). The results showed that 657 of the above 846 candidate transcripts were finally clustered into six modules, each of which showed a significant positive correlation with the ginsenoside Ro content (Figure 5c).

### 3.6. Visualization of Co-Expression Network and Mining of Hub Transcripts

**Figure 6:**
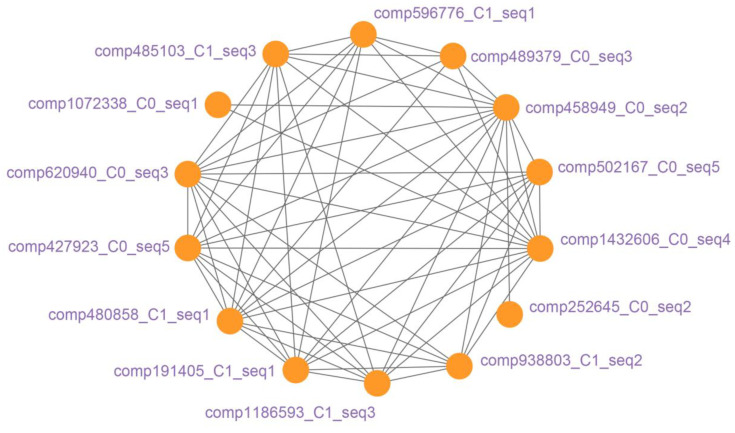
Co-expression network of 14 hub transcripts. Each orange node represents a hub transcript, the label next to each node is the name of the hub transcript, and each black edge represents the co-expression relationship between the two core transcripts it connects.

The results of the co-expression network visualization showed that the co-expression relationships between 657 candidate transcripts in the six modules were very close and formed a co-expression network including more than 180,000 edges. In the process of screening candidate transcripts within the network using weight values as indicators, and when the weight interval was adjusted to a high of 50%, there were still 462 candidate transcripts with 6464 edges in the network (Figure S2); when the weight interval was adjusted to a high of 15%, there were 14 candidate transcripts with 59 edges in the network (Figure 6). These 14 candidate transcripts were defined as hub transcripts.

The Spearman correlation coefficient-based interaction network was constructed using the expression (TPM) of these hub transcripts and the key enzyme transcripts reported to be directly involved in the synthesis of the ginsenoside Ro precursors, beta-Amyrin and oleanolic acid. This interaction network contained 136 edges and all transcripts were concentrated in one cluster (*p* value = 0.005), indicating that the protein encoded by the hub transcripts or those selected key enzyme transcripts were closely related and presumably interacted with each other in the biosynthesis of ginsenoside Ro (Figure S3).

### 3.7. Synteny Block and Phylogenetic Analysis of UGT Transcripts in Hub Transcripts

**Figure 7:**
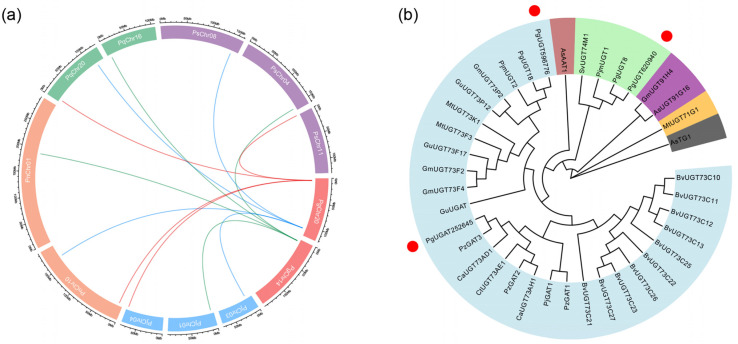
Synteny block and phylogenetic tree of *UGT* transcripts from hub transcripts: (**a**) synteny block of *UGT* transcripts within the genomes of different plants of the Ginseng genus. Rectangles in red, purple, green, orange and blue represent chromosomes representing chromosomes from *P. ginseng*, *P. stipuleanatus*, *P. quinquefolius*, *P. notoginseng* and *P. japonicus*, respectively. Arcs in red, blue, and green represent the synteny relationship of homologous sequence in different genomes of *PgUGT596776*, *PgUGAT252645* and *PgUGT620940*. Extrachromosomal scale represents the length of chromosome (Mb); (**b**) phylogenetic tree of *UGT* transcripts. Thirty-five enzyme proteins encoded by UGTs from *Avena strigose*, *Barbarea vulgaris*, *Carthamus tinctorius*, *Centella asiatica*, *Glycine max*, *Glycyrrhiza uralensis*, *Gypsophila vaccaria*, *Medicago truncatula*, *Panax ginseng*, *Panax japonicus* and *Panax zingiberensis* are capable of catalyzing the glycosylation modification of pentacyclic triterpenes. The amino acid sequences of these enzyme proteins were used for the constructing of the phylogenetic tree. Different colored branches represent different subfamilies of *UGTs*. The red dots mark the *UGT* transcripts among the hub transcripts in this work.

Three of the hub transcripts were functionally annotated with *UGTs* as a result, and the result of the synteny block analysis showed that the genes of these *UGT* transcripts are rearranged and mutated to some extent in the genomes of different plants of the *Panax L.* as the species has diverged, but highly homologous genes are retained in the genomes of these plants (Figure 7a). By phylogenetic analysis, we also found that the amino acid sequences encoded by *comp620940_C0_seq3* (*PgUGT620940*) and *comp596776_C1_seq1* (*PgUGT596776*) matched exactly with the two *UGTs* from *P. ginseng* (*PgUGT8* and *PgUGT18*) [17], which are directly involved in ginsenoside Ro synthesis. The amino acid sequence encoded by *comp252645_C0_seq2* (*PgUGAT252645*) showed 98.19% homology to a glucuronosyltransferase from *Panax zingiberensis* (*PzGAT3*) [19] that specifically modifies the C3 position of oleanolic acid, with no mutations in the conserved amino acids located in the PSPG (plant secondary product glucosyltransferase) box at the C-terminal of the amino acid sequence (Figure 7b). Thus, it was hypothesized that *PgUGAT252645* is the mRNA of the uridine diphosphate glucuronosyltransferase from *P. ginseng*, which can specifically modify the C3 position of oleanolic acid during ginsenoside Ro synthesis.

### 3.8. Analysis on the Effect of Mutations on the Function of PgUGAT252645

**Figure 8:**
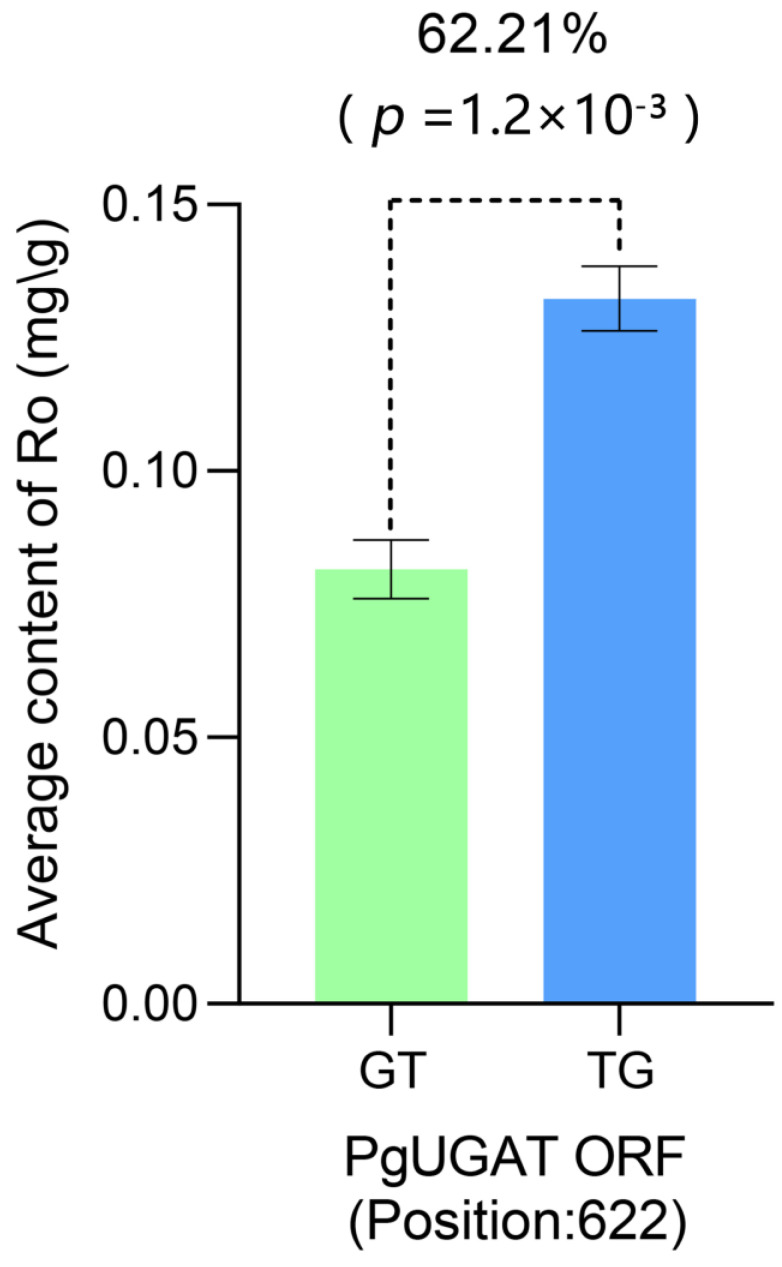
Genetic effects of SNP/InDel significantly associated with ginsenoside Ro content and located in the *PgUGAT* open reading frame on ginsenoside Ro content. The green and blue columns represent the mean values of ginsenoside Ro content in samples with different mutations at this SNP/InDel locus, respectively. Genetic effects as well as significance are labelled at the top.

During the synteny block analyses, we also identified SNP/InDels mutations in the open reading frame sequence of *PgUGAT252645* that result in amino acid changes (Figure S4). To ensure the rationality of the process of probing the functional impact of SNP/InDels mutations on *PgUGAT252645*, we mined SNPs/InDels in the UGT gene family using a total of 3943 UGT transcripts from the reference-free transcriptome database as the reference genome. A total of 9447 SNPs/InDels located in 1051 UGT transcripts were mined. After filtering and significance calculation, we identified 37 SNPs/InDels in 13 UGT transcripts with significant effects on the content of ginsenoside Ro (Table S1), including two consecutive base substitution mutations at 622 bp in the open reading frame of *PgUGAT252645* (Figure 8).

**Figure 9:**
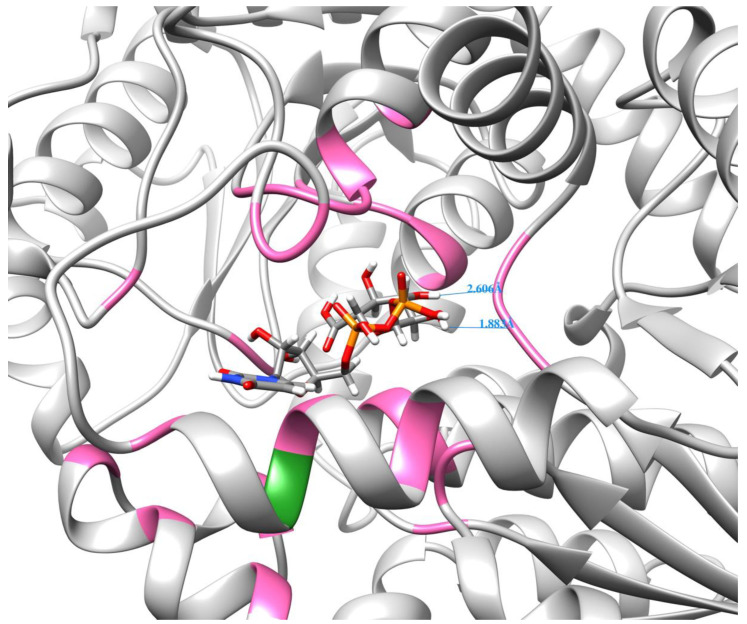
Molecular docking model of the protein encoded by *PGUGAT252645* gene with uridine diphosphate glucuronic acid as a glycosyl donor. Hydrogen bonds and their lengths in the model are marked with blue lines and text. Amino acids with colors (pink and green) in the peptide chain are amino acids located within 5 Å of the glycosyl donor molecule, where the amino acid in green is a cysteine replacing valine caused by the SNP/InDel described above.

Molecular docking analysis showed that the protein encoded by *PgUGAT252645* appears to bind with sugar donors better than the protein encoded by another UGAT transcript, *PgUGAT467663*, that is highly homologous to *PgUGAT252645* but differs from *PgUGAT252645* at 622 bp in its open reading frame. The docking model of *PgUGAT252645* not only has a lower binding free energy score, but the presence of two hydrogen bonds located between the glucuronide group and the protein means that the binding conformation of the protein to the sugar donor has higher specificity and stability (Figure 9).

In this docking model, the amino acid mutation resulting from the two consecutive base substitution mutations described above is located near the binding site (within 5 Å of the sugar donor). Thus, we speculate that it may be due to the fact that this amino acid mutation alters the spatial structure near the binding site to some extent, allowing for the sugar donor molecule to bind to the enzyme in a better position and thus improving the catalytic activity.

### 3.9. In Vitro and In Vivo Functional Validation of PGUGAT252645

**Figure 10:**
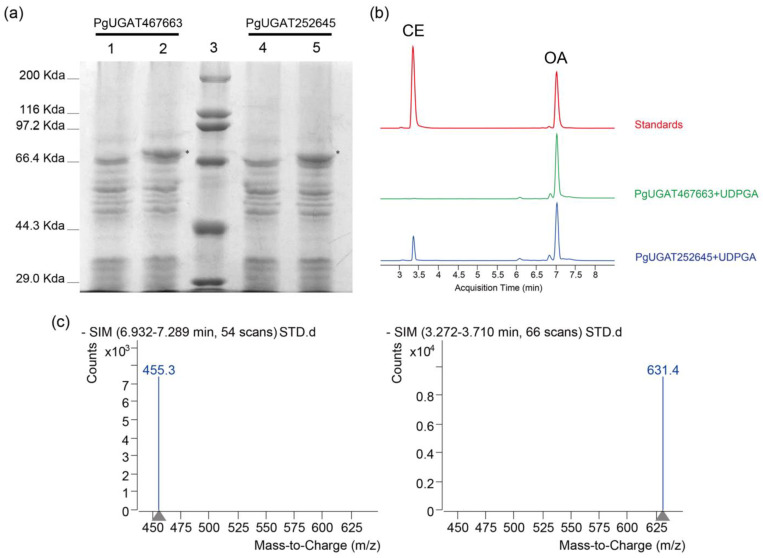
In vitro activity assay: (**a**) SDS-PAGE electrophoresis was used to detect the induced expression of recombinant proteins. Lanes 1 and 4 are uninduced samples. Lanes 2 and 5 are induced samples. Lane 3 is the protein molecular mass marker. The positions of recombinant proteins are marked by *; (**b**) liquid chromatographic analysis of the standard and extract samples. The red curve represents the standard solution containing oleanolic acid (OA) and Calenduloside E (CE). The green and blue curves represent the extract samples of different recombinant protein-catalyzed reaction systems, respectively; and (**c**) mass spectrometry detection of oleanolic acid standard (precursor ion mass: 455.3) and calenduloside E standard (precursor ion mass: 631.4).

To verify the results of the above analysis, we induced the expression of *PgUGAT467663* as well as *PGUGAT252645* using the *E. coli* expression system, respectively (Figure 10a, Figure S6). Then tested them for in vitro catalytic activity using oleanolic acid as the glucuronide-based acceptor and uridine diphosphate glucuronic acid (UDPGA) as the glucuronide-based donor. The results showed that the oleanolic acid C3 glucuronidation modification product, Calenduloside E, was detected in the reaction system containing the *PGUGAT252645* recombinant protein, whereas the corresponding glucuronidation product was not detected in the group of reaction systems containing the *PgUGAT467663* recombinant protein (Figure 10b,c).

To further validate the function of *PGUGAT252645*, we completed the ginseng adventitious roots genetic transformation operation of *PGUGAT252645* as well as *PgUGAT467663*, respectively, by the methods described previously (Figure S5). After the steps of culture, validation, and subsequent screening of transformed ginseng adventitious roots, we obtained seven and five hairy root asexual single root lines overexpressing *PGUGAT252645* or *PgUGAT467663* with stable growth status, respectively. We then examined the transcript levels of *PGUGAT252645* or *PgUGAT467663* and the contents of ginsenoside Ro in cultures of each hairy root asexual root line, using ginseng hairy roots induced by *Ag. rhizogenes* C58C1 carrying plasmid pCAMBIA1300-35S as a control.

**Figure 11:**
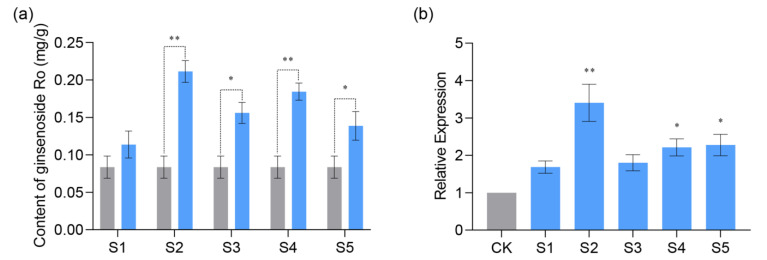

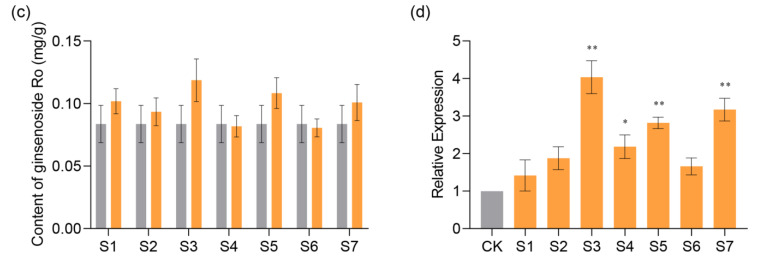
In vivo functional validation of *PGUGAT252645 gene*: (**a**,**b**) the blue columns represent the ginsenoside Ro content of the ginseng hairy roots overexpressing and the relative expression of *PGUGAT252645* gene; (**c**,**d**) the orange columns represent the ginsenoside Ro content and the relative expression of *PgUGAT467663* gene in overexpressing ginseng hairy roots; and (**a**,**d**) the grey columns represent the ginsenoside Ro content and the expression of the internal reference gene in the control ginseng hairy roots, respectively. The significance of the difference is expressed by “*”, “*” represents a significant difference, and “**” represents an extremely significant difference.

The results of the qRT-PCR showed that the transcript levels of *PGUGAT252645* were increased to varying degrees in all samples compared with the control, with a range of 1.509–3.967-fold (Figure 11a). Meanwhile, the ginsenoside Ro content was significantly higher in four of the five samples overexpressing *PgUGAT252645* compared to the control, and the trend correlated with the degree of enhanced expression (Figure 11b). Although the ginsenoside Ro content of some of the samples overexpressing *PgUGAT467663* was also enhanced, the increase was generally much smaller than that of the samples overexpressing *PgUGAT252645* and was not related to the degree of enhanced expression (Figure 11c,d). These results validated our speculation on the function of *PgUGAT252645*.

## 4. Discussion

In order to improve the ginsenoside content of *P. ginseng*, several genes from different gene families capable of affecting the synthesis of ginsenosides were identified and analyzed, such as, *bHLHs* [36], *bZIPs* [37], *P450s* [38], *GRASs* [39], and *MYBs* [40]. With the development of synthetic biology, the heterologous synthesis of ginsenosides in microbial cell factories offers a sustainable solution [41,42,43,44]. To this end, several genes derived from multiple species directly involved in the synthesis of ginsenoside or intermediates were cloned and characterized. They were then optimally modified and transferred into the host microbial cell to participate in the pathway within the host microbial cell that was artificially designed for controlled ginsenoside synthesis [45,46,47].

Although some progress has been made on the genomic level of *P. ginseng* [30,48], the genomic structure of *P. ginseng* is complex. The expression of genes from *P. ginseng* has spatial and temporal specificity [49], which led to difficulties in mining certain genes from *P. ginseng*, such as the genes encoding uridine diphosphate glycosyltransferases (*PgUGTs*), a group of enzymes that can catalyze glycosylation modifications of ginsenosides. In early works, genes identified as encoding *PgUGTs* were transferred to *Escherichia coli* for in vitro expression, and the activity of the expression product against specific substrates was used as an important reference for the function of the gene [50]. With the development and availability of sequencing technology, more *PgUGTs* genes in *P. ginseng* have been discovered. For their functional prediction, in addition to in vitro expression validation, homology with the functionally validated *PgUGTs* and the correlation between their expressions and ginsenoside content will be used as a reference [51].

When the mining target is not limited to members of a particular gene family, a more efficient approach of screening and functional validation is required. In this work, 846 candidate transcripts were screened by differential expression analysis from over 20,000 transcripts that showed significant positive correlations with ginsenoside Ro content. Then, a weighted co-expression network of these candidate transcripts was established by combining the phenotypic data of ginsenoside Ro content and 14 hub transcripts were screened in this network using the weight values as indicators. Finally, we used the results of functional annotation and sequence alignment as references to identify three *PgUGTs* from hub transcripts. Among them, the functional verification results of the unreported *PgUGAT252645* confirmed our speculation that *PgUGAT252645* was directly involved in the synthesis of ginsenoside Ro. In this process, because of the construction of a co-expression network, the efficiency and accuracy of gene screening and verification were improved to a certain extent.

It is noteworthy that among the 14 hub transcripts finally screened, the amino acid sequence encoded by the open reading frame of *comp1186593_C1_seq3* exactly matched the amino acid sequence of the reported cellulose synthase-like glucosyltransferase *PgCSyGT1* [17]. Although *PgUGAT252645* and *PgCSyGT1* belong to different gene families, they may perform similar functions in the process of ginsenoside Ro synthesis. Accordingly, we speculate that, for the glycosylation modification step in ginsenoside synthesis, there is not only a hybrid catalytic phenomenon in which a single glycosyltransferase can catalyze multiple substrate glycosylation modifications, but also a phenomenon in which multiple glycosyltransferase isozymes catalyze a substrate glycosylation modification together, and these glycosyltransferase isozymes may come from different gene families. Just as studies of substrate preference can elucidate the main pathways in the metabolic network of ginsenoside glycosylation modification with hybrid catalysis, the exploration of the relationship between these isoenzymes and the deeper mining of other isoenzyme members will further elucidate the ginsenoside synthesis process.

In addition to the glycosyltransferases, the hub transcripts also include members of the common plant secondary-metabolism regulatory factors, the *MYB*, *bHLH*, and *ERF* gene families, as well as members of the *GRF* and *ARF* gene families involved in growth and development-related signal transduction pathways [52]. Based on this, it is speculated that the hub transcripts may include regulatory genes before the synthesis of ginsenoside Ro, genes directly involved in the synthesis process, and response genes after synthesis. The functions of these hub transcripts are also of great research value, and further research of them is of great significance for revealing the synthesis mechanism and metabolic pathways of ginsenoside Ro.

## 5. Conclusions

After differential expression analysis and weighted co-expression network analysis of 22,481 transcripts from the newly constructed *P. ginseng* reference-free transcriptome database, which showed significant positive correlation with ginsenoside Ro content, 14 hub transcripts were selected, including *PgUGAT252645*, whose coding region is highly homologous to the reported uridine diphosphate glucuronosyltransferase from *Panax zingiberensis* (*PzGAT3*). Results of the functional analysis and activity validation suggest that the protein encoded by *PgUGAT252645* may perform the same function as the reported *PgCSyGT1* in catalyzing the glucuronidation modification of oleanolic acid during ginsenoside Ro synthesis. These results provide new information and knowledge on the technical basis and theoretical rationale required for the work of ginsenoside synthesis-related genes and further reveal the mechanism of ginsenoside synthesis.

## Data Availability

The datasets generated during and analyzed during the current study and all plant materials are available from the corresponding author on reasonable request.

## Supplementary Materials

The following supporting information can be downloaded at: https://www.mdpi.com/article/10.3390/plants13050604/s1, Figure S1: Histogram of transcript length distribution within the un-referenced transcriptome; Figure S2: Weighted gene co-expression network of candidate transcripts; Figure S3: Interaction network of hub transcripts with transcripts of ginsenoside Ro synthesis key enzyme genes; Figure S4: Comparison of the amino acid sequence encoded by *PgUGAT252645* and another transcript *PgUGAT467663* under the same gene ID; Figure S5: Induction and culture of ginseng hairy roots; Figure S6. Full-length gel and blots of SDS-PAGE of protein induction; Table S1: SNPs/InDels with significant effects on the content of ginsenoside Ro; Table S2: Primers used in this study; Table S3: LC-MS conditions for the analysis of in vitro enzyme activity detection; Table S4: RT-PCR reaction conditions; Table S5: LC-MS conditions for the analysis of ginsenoside Ro content in *P. ginseng* hairy roots; Table S6: Information of genome and proteins for Synteny block and phylogenetic analysis.
